# Long-term Impact of Childhood Adiposity on Adult Metabolic Syndrome Is Modified by Insulin Resistance: The Bogalusa Heart Study

**DOI:** 10.1038/srep17885

**Published:** 2015-12-07

**Authors:** Huijie Zhang, Tao Zhang, Shengxu Li, Ying Li, Azad Hussain, Camilo Fernandez, Emily Harville, Lydia A. Bazzano, Jiang He, Wei Chen

**Affiliations:** 1 Department of Endocrinology and Diabetes, The First Affiliated Hospital, Xiamen University, Xiamen, China; 2 Tulane Center for Cardiovascular Health and Department of Epidemiology, Tulane University Health Sciences Center, New Orleans, LA; 3 Department of Biostatistics, School of Public Health, Shandong University, Jinan, China; 4 Department of Nutrition and Food Hygiene, School of Public Health, Harbin Medical University, Harbin, China

## Abstract

Childhood adiposity and insulin resistance are well-known risk factors for adult metabolic syndrome (MetS). This study aims to examine whether the association between childhood adiposity and adult MetS is modified by insulin resistance. The cohort consisted of 1,593 black and white subjects, aged 19–50 years at follow-up, who were examined 19 years apart on average as children and adults for MetS variables. The prevalence of adult MetS was compared between the insulin-sensitive obesity and insulin-resistant obesity groups in childhood. Adult MetS prevalence was higher in the insulin-resistant obesity group than in the insulin-sensitive obesity group (34.9% vs. 24.3%, p = 0.008). In multivariable logistic regression analyses adjusted for age, race, gender, and follow-up years, individuals with insulin-resistant obesity in childhood were 1.7 times (p = 0.011) more likely to have MetS 19 years later on average than those with insulin-sensitive obesity in childhood. Odds ratio did not differ significantly between blacks and whites (p = 0.724). ORs for the association of childhood BMI with adult MetS significantly increased with increasing tertiles of childhood HOMA (p < 0.001 for trend). These findings suggest that insulin resistance amplifies the association between childhood adiposity and adult MetS and underscore the importance of preventing both adiposity and insulin resistance in early life.

Metabolic syndrome (MetS) is considered a cluster of multiple interrelated metabolic disorders, including obesity, insulin resistance, dyslipidemia, hypertension, and hyperglycemia^1^,^2^. MetS has gained importance because of its close association with type 2 diabetes and cardiovascular disease^3^,^4^. It has been well documented that adiposity and insulin resistance are strongly associated with the development of MetS in the general population^5^,^6^. Obesity is highly correlated with insulin resistance; thus, it may play a key role as an initiating factor in the development of MetS^3^,^7^. Evidence from epidemiological studies has indicated that the origins of chronic diseases, including type 2 diabetes and MetS, begin early in life, even in utero^8^, and both childhood adiposity and insulin resistance are well-established risk factors for adult diabetes, MetS and cardiovascular disease^9^,^10^,^11^. Investigation of the childhood origins of MetS has implications for preventive measures of MetS in early life.

It has been proposed that the association between obesity and MetS is more than merely a dose-response relationship^12^,^13^,^14^. Insulin resistance is considered a key link between obesity and type 2 diabetes or MetS. Recent studies have demonstrated that some obese adults and children with higher insulin sensitivity exhibit a relatively better metabolic profile compared to those with insulin-resistant obesity^15^,^16^,^17^,^18^,^19^. Longitudinal studies have indicated that insulin-sensitive obese individuals may be protected from increased risk of type 2 diabetes and cardiovascular disease^16^,^20^,^21^. Furthermore, studies have shown that the presence of metabolic disorders varies widely among obese individuals as a function of differences in the degree of insulin sensitivity^12^,^18^,^22^. Despite extensive observations on insulin-resistant and sensitive obesity, information is scant regarding whether the association between childhood adiposity and MetS in later life is dependent on insulin resistance. In the current study, we aimed to test the hypothesis that the effect of early-life adiposity on the development of MetS is modified by insulin resistance in childhood in a longitudinal cohort from the Bogalusa Heart Study.

## Results

Table 1 summarizes the mean levels of study variables in childhood and adulthood by race and gender. In childhood, age and BMI did not significantly differ between races or genders. There were significant race differences in childhood glucose (whites > blacks) and insulin levels (blacks > whites). Gender differences were also significant in childhood glucose (boys > girls) and insulin levels (girls > boys). Black children had higher HOMA values than white children (2.5 vs. 2.3, p = 0.006). In adulthood, blacks had higher levels of BMI, glucose, insulin, blood pressure, HOMA, and HDL-C, and lower levels of triglycerides than whites. Black women had higher WC than white women, but white men had higher WC than black men. At follow-up, the prevalence of MetS did not show a significant race difference (18.2% in blacks vs. 23.0% in whites, p = 0.127).

**Figure 1:**
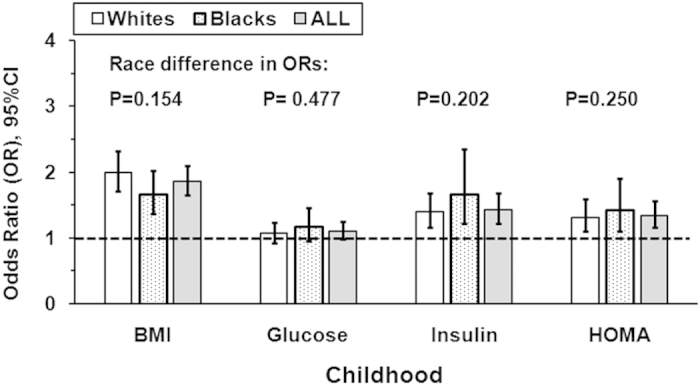
Odds ratio (OR) for the association of childhood BMI, glucose, insulin and HOMA with adult MetS by race, adjusted for covariates of age, gender, follow-up years or race. HOMA = homeostasis model assessment of insulin resistance; BMI = body mass index.

Figure 1 shows odds ratios (OR) for the associations of childhood BMI, glucose, insulin and HOMA levels with adult MetS by race. After adjusting for age, gender, and follow-up years, childhood BMI, insulin (log-transformed) and HOMA (log-transformed) all showed significant associations with adult MetS in blacks and whites, but childhood glucose did not show a significant association. There were no race differences in ORs for the associations of childhood BMI, glucose, insulin and HOMA with adult MetS (all p > 0.05).

As shown in Supplement Table S1, childhood log-HOMA was significantly associated with adult overweight/obesity (OR = 1.70, p < 0.001), hyperglycemia (OR = 1.35, p = 0.001), high blood pressure (OR = 1.28, p < 0.001) and dyslipidemia (OR = 1.23, p < 0.001), adjusting for baseline age, race, gender, and follow-up years.

Mean levels of study variables by levels of childhood BMI and HOMA values are shown in Table 2. After adjusting for age, race, gender and follow-up years, high levels of childhood BMI and HOMA were significantly associated with higher levels of adult MetS variables, including BMI, WC, insulin, blood pressure, HOMA, and prevalence of MetS. The insulin-resistant obesity group had significantly higher levels of BMI, WC, insulin, and systolic blood pressure in adulthood than the insulin-sensitive obesity group, adjusting for race, gender, childhood age and follow-up years. Adult levels of HOMA and diastolic blood pressure were marginally higher in the insulin-resistant obesity group than in the insulin-sensitive obesity group, but serum glucose, HDL-C, and triglycerides in adulthood did not showed significant difference between the two groups. Of note, prevalence of MetS was significantly higher in the insulin-resistant obesity group than the insulin-sensitive obesity group (34.9% vs. 24.3%, p = 0.008).

Multivariable-adjusted ORs for the association between childhood insulin-resistant adiposity and adult MetS, using insulin-sensitive adiposity as a reference, are presented in Table 3. Individuals with insulin-resistant obesity were 1.7 times (p = 0.011) more likely to have MetS than those with insulin-sensitive obesity, after adjustment for baseline age, race, gender, and follow-up years. The ORs did not differ significantly (p = 0.724 for race difference) between whites (OR = 1.72, p = 0.026) and blacks (OR = 1.63, p = 0.206). The follow-up period ranged from 5.0 to 33.8 years. Logistic regression models performed by tertile of follow-up years yielded ORs (95% CIs) that were 2.50 (1.00–7.15), 1.75 (0.86–3.72) and 1.47 (0.81–2.67) in the first, second and third tertile of follow-up years (5.0–14.6, 14.7–23.6 and 23.7–33.8 years), respectively. The ORs did not differ significantly between groups.

The multivariable-adjusted ORs for the association between adult MetS and childhood insulin-resistant and insulin-sensitive adiposity, defined by insulin or glucose levels, are shown in Supplement Table S2. Individuals with childhood adiposity who also had high insulin levels were 1.53 times (p = 0.036) more likely to have MetS than those with childhood adiposity who had lower insulin levels; the association did not differ between whites (OR = 1.68, p = 0.032) and blacks (OR = 1.20, p = 0.636). In contrast, childhood adiposity with high glucose levels was not associated with adult MetS.

**Figure 2:**
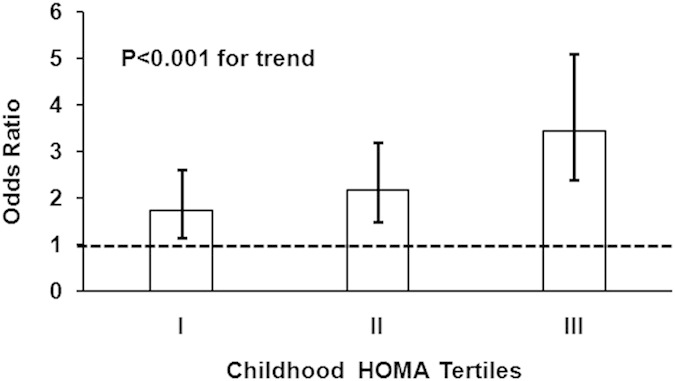
Odds ratio for the association of childhood BMI tertiles with adult MetS by childhood HOMA tertile groups, adjusted for covariates age, race, gender, and follow-up years. HOMA = homeostasis model assessment of insulin resistance. BMI = body mass index.

Figure 2 shows the ORs for the association of childhood BMI tertiles with adult MetS by childhood HOMA levels (tertiles). Of note, the OR values for the association of childhood BMI with adult MetS significantly increased across the increasing tertiles of childhood HOMA levels (OR = 1.73 in tertile I, OR = 2.18 in tertile II, OR = 3.45 in tertile III, p < 0.001 for trend). The significant increasing trend in ORs reflects the interaction between childhood adiposity and insulin resistance on the subsequent occurrence of adult MetS. Additionally, the interaction effect of childhood BMI with HOMA on adult MetS was also significant (p = 0.006) using HOMA as a continuous variable, which was substantially similar to the interaction effect using HOMA tertiles.

## Discussion

Obesity and insulin resistance are well-known risk factors for MetS in children and adults^9^,^23^,^24^. By investigating the childhood origins of MetS, future preventive measures in early life can be appropriately targeted. We have previously reported that childhood BMI and insulin predict adulthood MetS^25^. However, previous data did not examine the role of insulin resistance in the relationship between adiposity in early life and the development of MetS in later life. In the current study, individuals with insulin-resistant obesity in childhood were more likely to develop adult MetS than those with insulin-sensitive obesity in childhood. This study provides strong evidence that the effect of childhood adiposity on the development of adult MetS is modified by insulin resistance in childhood. These observations suggest that in the presence of insulin-resistance, the association between childhood adiposity and MetS in later life is strengthened.

Childhood adiposity has been observed to track into adulthood, and is recognized as a predictor of MetS in later life^26^,^27^. In a review of 11 studies investigating the association between childhood BMI and adult metabolic risk, childhood adiposity was positively associated with total cholesterol, LDL-C, triglyceride and insulin concentration in adulthood^28^. Three studies reported positive correlations between childhood BMI and the number of MetS criteria and risk of developing MetS in adulthood^11^,^25^,^29^. Morrison *et al.* reported that the OR for developing MetS in adulthood was 1.03 with each one unit increase in childhood BMI percentile^29^. We noted in this study that early life adiposity along with higher insulin and HOMA levels during childhood was strongly associated with increased risk of MetS in adulthood.

Insulin resistance is closely associated with obesity and considered to play a pathogenic role in MetS^24^,^30^. A subset of obese individuals are often described as metabolically healthy obese and are characterized by high insulin sensitivity which may protect them to some extent against obesity-related metabolic complications^13^. Vukovic *et al.* reported that obese children who were insulin-sensitive have significantly “healthier” metabolic profiles compared to their insulin-resistant peers in a group of 248 children and adolescents^19^. Karelis and colleagues reported insulin-sensitive obese individuals had a lower levels of inflammatory markers compared with those with insulin resistance among 88 obese postmenopausal women^14^. Longitudinal studies have also suggested that these individuals have a lower risk of type 2 diabetes and cardiovascular disease than their insulin-resistant counterparts^16^,^20^. In the present study, we found that prevalence of adult MetS was significantly higher in the insulin-resistant obesity group than in the insulin-sensitive obesity group. This study expands previous findings by demonstrating that the relationship between childhood obesity and adult MetS is modified by childhood insulin resistance. In the current study, we found that individuals with insulin-resistant obesity in childhood were 1.7 times more likely to have MetS 19 years later on average than those with insulin-sensitive obesity. More importantly, the strength of the association significantly increased across increasing tertiles of childhood HOMA values.

In addition, we found that blacks had higher HOMA than whites during childhood and adulthood in the present study; however, the strength of the association between childhood BMI and adult MetS across levels of childhood HOMA values did not differ between races. To the best of our knowledge, this is one of the first studies to examine the childhood origins of adult MetS in black and white individuals and further examines the interplay of adiposity and insulin resistance. Our data indicated that insulin resistance amplified the association between childhood adiposity and adult MetS equally in both races. These observations across race groups should be confirmed and replicated in other population studies.

There are several putative mechanisms linking childhood obesity and insulin resistance to MetS in later life. First, obesity tends to persist from childhood into adulthood. The Bogalusa Heart Study and other studies have found that the tracking of obesity across the lifespan contributed to a higher risk of metabolic disorders in adulthood^28^,^31^. This tracking phenomenon may contribute to the association between childhood obesity and adult MetS. Second, obese individuals with insulin resistance might have additional predisposing factors as compared to insulin-sensitive obese individuals, for example higher lipid deposition in intramyocellular and visceral compartments and lower adiponectin levels^15^,^32^. Under these conditions, insulin resistance may amplify adverse effects of childhood adiposity on metabolic profile in adulthood. Third, genetic susceptibility may contribute to the interplay of obesity and insulin resistance on the development of MetS. Previous studies have indicated that decreased expression of genes involved in glucose uptake and lipogenesis in adipose tissue was associated with insulin-resistant obesity^33^,^34^,^35^.

This community-based, longitudinal cohort provided a unique opportunity to test the effect insulin-resistance on the relationship between early-life adiposity and the development of MetS in adulthood. The present study has some limitations. First, categorizing childhood adiposity and insulin resistance as the upper 55^th^ percentile of BMI and HOMA, respectively, seems broad. The relatively small sample size and consequently limited statistical power was the primary consideration for the selection of the cut-off values. In sensitivity analyses using a cut-off value of 75^th^ percentile and above for BMI and HOMA, ORs remained statistically significant. Second, the follow-up period of the study subjects varied from 5 to 34 years. The wide range of follow-up time might have an influence on the obesity/insulin resistance-MetS association. The number of follow-up years was, therefore, included in all logistic models for adjustment in the current analysis. Moreover, we conducted additional analysis by tertiles of follow-up year and did not find a significant difference in ORs between groups.

In conclusion, we demonstrated that childhood adiposity with insulin resistance was associated with an increased risk of MetS in later life as compared to those with insulin-sensitive obesity in childhood. In particular, ORs for the association of childhood BMI with adult MetS significantly increased with across tertiles of childhood HOMA. This significant increasing trend reflects the impact of insulin resistance on the relationship between childhood adiposity and adult MetS. These results support the notion that the long-term impact of childhood adiposity on the development of MetS in later life begins in childhood, and is further amplified by insulin resistance. These findings underscore the importance of controlling both adiposity and insulin resistance in early life for the prevention of MetS later in life.

## Methods

### Study cohort

The Bogalusa Heart Study is a series of long-term studies in a semi-rural biracial (65% white and 35% black) community in Bogalusa, Louisiana begun in 1973 by Dr. Gerald Berenson, focusing on the early natural history of cardiovascular disease since childhood. Between 1973 and 2010, nine cross-sectional surveys of children aged 4–19 years and ten cross-sectional surveys of adults aged 19–50 years who had been previously examined as children were conducted in Bogalusa. Linking these repeated cross-sectional examinations conducted every 2–3 years has resulted in serial observations during childhood. A total of 1,593 adult subjects (1,032 whites and 561 blacks; 42.6% males; age range = 19–50 years; mean age = 31.5 years at follow-up) who were examined for MetS variables as both children and adults, with a mean follow-up period of 19.2 years (range = 5.0–33.8 years), were included in the present analysis. For individuals who participated in multiple screenings as children and adults, data from the earliest childhood and most recent adulthood examination were used to maximize the follow-up interval. All subjects in this study gave informed consent at each examination, and for those under 18 years of age, consent of a parent/guardian was obtained. Study protocols were approved by the Institutional Review Board of the Tulane University Health Sciences Center. The methods were carried out in accordance with the approved guidelines.

### General examinations

Standardized protocols were used by trained examiners across all surveys since 1973^36^. Subjects were instructed to fast for 12 hours before screening. Body mass index (BMI, weight in kilograms divided by the square of the height in meters) was used as a measure of adiposity. The waist circumference was measured midway between the rib cage and the superior border of the iliac crest while subjects were standing. Blood pressure levels were measured between 8:00 AM and 10:00 AM on the right arm of subjects in a relaxed, sitting position by 2 trained observers (3 replicates each). Systolic and diastolic (4th Korotkoff phase for children and 5th Korotkoff phase for adults) blood pressure was recorded using a mercury sphygmomanometer^37^. The mean values of the 6 readings were used for analysis. Three measurements were obtained with a nonstretchable tape, and the mean value was used for analysis.

### Laboratory analyses

Serum triglycerides (TG) levels were assayed using enzymatic procedures on a Hitachi 902 Automatic Analyzer (Roche Diagnostics, Indianapolis, IN). Serum lipoprotein cholesterol levels were analyzed by a combination of heparin-calcium precipitation and agar-agarose gel electrophoresis procedures. A commercial radioimmunoassay kit was used for measuring plasma immunoreactive insulin levels (Phadebas; Pharmacia Diagnostics, Piscataway, NJ). Plasma Glucose levels were measured as part of a multiple chemistry profile (SMA20; Laboratory Corporation of America, Burlington, NC) by a glucose oxidase method. Insulin resistance status was assessed using the homeostasis model assessment of insulin resistance (HOMA) according to the formula described previously: HOMA = insulin (μU/mL) × glucose (mmol/L)/22.5.

### Statistical methods

All statistical analyses were performed with SAS version 9.3 (SAS Institute, Cary, NC). Adult MetS was defined according to NCEP ATPIII guidelines^38^. These criteria included: 1) abdominal obesity (waist circumference ≥102 cm for men and ≥88 cm for women), 2) hypertriglyceridemia (fasting triglycerides ≥ 150 mg/dL), 3) low HDL cholesterol (<40 mg/dL for men and <50 mg/dL for women, or taking cholesterol lowering medication), 4) high blood pressure (≥130/85 mmHg or taking antihypertensive medication), and 5) high fasting glucose (≥100 mg/dL or taking antidiabetic medication) (24). Childhood fasting glucose and insulin values were used to calculate HOMA. Childhood adiposity and insulin resistance were defined as being above 55^th^ percentile of BMI and HOMA, respectively, with 10% of children in the middle (46^th^ – 55^th^ percentile) excluded to make the groups more distinct. The exclusion of this 10% of children for BMI and HOMA values resulted in an exclusion of 19.1% of the total sample. Accordingly, the remaining cohort was divided into 4 groups: group I (BMI↓/HOMA↓), group II (BMI↓/HOMA↑), group III (BMI↑/HOMA↓), and group IV (BMI↑/HOMA↑). The prevalence of adult MetS was compared between the childhood insulin-sensitive obesity group (group III, n = 235) and the childhood insulin-resistant obesity group (group IV, n = 416). Analyses of covariance were performed using general linear models (GLM) to test differences in study variables between race, gender and adiposity groups. Multivariable logistic regression models were used to examine the association between adult MetS and insulin-resistant obesity, adjusted for baseline age, race, gender, and follow-up years. The modification effect of childhood insulin resistance on the BMI-MetS association was examined by logistic regression interaction models using the entire sample. Because the wide range of follow-up years might have an impact on ORs for the association between obesity/insulin resistance and MetS, logistic regression models were also performed by tertile of follow-up years.

## Additional Information

**How to cite this article**: Zhang, H. *et al.* Long-term Impact of Childhood Adiposity on Adult Metabolic Syndrome Is Modified by Insulin Resistance: The Bogalusa Heart Study. *Sci. Rep.* **5**, 17885; doi: 10.1038/srep17885 (2015).

## Supplementary Material

Supplementary Information

## Figures and Tables

**Table 1:** Population characteristics in childhood and adulthood by race and gender.

	Whites		Blacks		P-values^a^	
	Males	Females	Males	Females	Males	Females
Sample size	458	574	220	341		
Childhood						
Age (year)	12.3 (3.6)	11.8 (3.5)	11.8 (3.8)	11.8 (3.8)	0.012	0.991
BMI (kg/m^2^)	19.5 (4.1)	19.0 (4.3)	19.1 (4.5)	19.4 (4.4)	0.966	0.146
Glucose (mg/dL)	84.8 (7.1)**	82.6 (7.6)	83.1 (9.1)**	80.9 (8.4)	0.015	0.001
Insulin (μU/mL)	10.4 (7.0)**	11.4 (7.9)	11.0 (9.5)*	13.0 (8.7)	0.114	0.003
HOMA	2.2 (1.6)*	2.4 (1.7)	2.3 (2.2)	2.6 (1.9)	0.207	0.017
Adulthood						
Age (year)	31.9 (8.0)	31.3 (8.1)	30.2 (8.8)	30.4 (8.1)	0.012	0.091
Follow-up years	19.6 (7.1)	19.5 (7.0)	18.5 (7.7)	18.6 (7.4)	0.054	0.049
WC (cm)	96.4 (17.1)**	85.2 (17.8)	92.1 (18.9)	91.1 (18.4)	0.033	<0.001
BMI (kg/m^2^)	28.4 (6.2)*	27.4 (7.7)	28.6 (7.4)**	30.4 (8.4)	0.237	<0.001
Glucose (mg/dL)^b^	85.9 (15.1)**	81.7 (9.8)	88.9 (24.7)**	84.0 (16.6)	0.003	0.001
Insulin (μU/mL)^b^	12.0 (9.3)	11.4 (8.7)	12.4 (9.3)*	14.5 (10.0)	0.378	<0.001
HOMA^b^	2.6 (2.4)	2.4 (2.1)	2.9 (2.7)	3.1 (2.4)	0.078	<0.001
Systolic BP(mmHg)^b^	115.3 (10.4)**	107.6 (9.5)	119.6 (15.0)**	111.8 (11.7)	<0.001	<0.001
Diastolic BP(mmHg)^b^	77.5 (8.5)**	72.7 (8.0)	78.1 (12.2)**	74.1 (9.8)	0.006	0.001
HDL-C (mg/dL)^b^	42.0 (11.0)**	49.0 (12.5)	51.8 (16.2)	53.2 (14.2)	<0.001	<0.001
Triglycerides (mg/dL)^b^	141.9 (111.3)**	111.2 (66.5)	109.1 (81.1)**	80.9 (37.1)	0.001	<0.001
MetS, n (%)	118 (25.8)	119 (20.7)	45 (20.5)	57 (16.7)	0.349	0.290

Continuous variables are presents as means (SD).

BMI = body mass index; HOMA = homeostasis model assessment of insulin resistance; WC = Waist circumference; BP = blood pressure; HDL-C = high-density lipoprotein cholesterol; MetS = metabolic syndrome.

Gender difference: *p < 0.05; **p < 0.01.

^a^P-values for race difference in continuous MetS variables were adjusted for age.

^b^Individuals who took medications were excluded.

**Table 2:** Group characteristics by levels of childhood BMI and HOMA.

	Groups by levels of childhood BMI and HOMA^a^				P-Value†	P-Value‡
	Group I: BMI↓/HOMA↓	Group II: BMI↓/HOMA↑	Group III: BMI↑/HOMA↓	Group IV: BMI↑/HOMA↑		
Sample size	398	239	235	416		
Whites/Blacks	263/135	149/90	146/89	276/140	0.554	0.319
Males/Females	174/224	101/138	87/148	184/232	0.303	0.087
Childhood						
Age (year)	12.4 (3.6)	12.5 (3.4)	12.1 (3.9)	11.9 (3.5)	0.064	0.393
BMI (kg/m^2^)	16.7 (2.0)	17.0 (2.0)	21.1 (3.7)	22.9 (4.9)	<0.001	<0.001
Glucose (mg/dL)	80.8 (7.3)	85.6 (8.5)	80.1 (7.6)	85.1 (7.4)	<0.001	<0.001
Insulin (μU/mL)	6.3 (2.2)	15.6 (7.6)	6.7 (2.5)	17.9 (9.8)	<0.001	<0.001
HOMA	1.3 (0.4)	3.3 (1.7)	1.3 (0.5)	3.8 (2.2)	<0.001	<0.001
Adulthood						
Age (year)	31.1 (8.2)	32.7 (8.1)	31.2 (8.3)	31.5 (8.2)	0.093	0.661
Follow-up years	18.7 (7.2)	20.2 (7.7)	19.1 (7.1)	19.7 (7.3)	0.056	0.349
WC (cm)	82.6 (14.4)	83.8 (14.3)	95.4 (18.3)	101.6 (19.2)	<0.001	<0.001
BMI (kg/m^2^)	24.7 (5.2)	25.0 (5.0)	31.3 (7.4)	33.5 (8.1)	<0.001	<0.001
Glucose (mg/dL)^b^	81.9 (9.8)	84.9 (18.6)	85.5 (20.0)	86.5 (18.1)	<0.001	0.656
Insulin (μU/mL)^b^	9.3 (5.9)	11.0 (7.8)	14.0 (12.1)	15.8 (10.8)	<0.001	0.049
HOMA^b^	1.9 (1.5)	2.5 (2.3)	3.1 (2.9)	3.5 (2.8)	<0.001	0.062
Systolic BP(mmHg)^b^	110.8 (11.0)	112.0 (10.9)	112.3 (11.3)	114.8 (13.2)	<0.001	0.031
Diastolic BP(mmHg)^b^	74.2 (8.6)	75.2 (9.1)	75.1 (8.5)	76.8 (10.6)	<0.001	0.090
HDL-C (mg/dL)^b^	49.7 (14.1)	51.3 (14.5)	48.0 (13.5)	45.6 (13.2)	<0.001	0.127
Triglycerides (mg/dL)^b^	100.3 (74.9)	107.6 (70.5)	113.9 (84.8)	125.2 (86.1)	<0.001	0.341
MetS, n (%)	45 (11.3)	35 (14.6)	57 (24.3)	145 (34.9)	<0.001	0.008

Continuous variables are presents as means (SD).

BMI = body mass index; HOMA = homeostasis model assessment of insulin resistance; WC = Waist circumference; BP = blood pressure; HDL-C = high-density lipoprotein cholesterol; MetS = metabolic syndrome.

^a^Low and high levels of childhood BMI and HOMA were defined as bottom and top 45^th^ percentiles, respectively.

^b^Individuals who took medications were excluded.

^†^P-values for difference in continuous MetS variables among 4 groups were adjusted for race, gender, and age.

^‡^P-values for difference in continuous MetS variables between groups III and IV were adjusted for race, gender, and age.

**Table 3:** Association between childhood insulin-resistant adiposity and adult MetS, adjusting for covariates in logistic regression models.

	Using childhood insulin-sensitive adiposity as a reference								
	Whites (n = 422)			Blacks (n = 229)			Total (n = 651)		
	OR	95%CI	P	OR	95%CI	P	OR	95% CI	P
Black race	—	—	—	—	—	—	0.69	0.46–1.03	0.074
Female sex	0.55	0.35–0.86	0.008	0.63	0.31–1.30	0.213	0.57	0.39–0.83	0.003
Childhood age	1.07	1.00–1.14	0.041	1.16	1.06–1.28	0.002	1.10	1.05–1.16	<0.001
Follow-up years	1.13	1.09–1.17	<0.001	1.15	1.10–1.21	<0.001	1.14	1.11–1.17	<0.001
Childhood insulin-resistant adiposity	1.72	1.08–2.80	0.026	1.63	0.77–3.56	0.206	1.69	1.13–2.54	0.011

OR = odds ratio; CI = confidence interval.

## References

[b1] Malik S. *et al.* Impact of the metabolic syndrome on mortality from coronary heart disease, cardiovascular disease, and all causes in United States adults. Circulation 110, 1245–1250 (2004). 15326067 10.1161/01.CIR.0000140677.20606.0E

[b2] Liu L. *et al.* Impact of metabolic syndrome on the risk of cardiovascular disease mortality in the United States and in Japan. Am J Cardiol 113, 84–89 (2014). 24169008 10.1016/j.amjcard.2013.08.042

[b3] Jensen M. D. *et al.* 2013 AHA/ACC/TOS guideline for the management of overweight and obesity in adults: a report of the American College of Cardiology/American Heart Association Task Force on Practice Guidelines and The Obesity Society. Journal of the American College of Cardiology 63, 2985–3023 (2014). 24239920 10.1016/j.jacc.2013.11.004

[b4] Aguilar M., Bhuket T., Torres S., Liu B. & Wong R. J. Prevalence of the metabolic syndrome in the United States, 2003–2012. JAMA 313, 1973–1974 (2015). 25988468 10.1001/jama.2015.4260

[b5] Goff D. C. Jr. *et al.* 2013 ACC/AHA guideline on the assessment of cardiovascular risk: a report of the American College of Cardiology/American Heart Association Task Force on Practice Guidelines. Journal of the American College of Cardiology 63, 2935–2959 (2014). 24239921 10.1016/j.jacc.2013.11.005 PMC4700825

[b6] Cornier M. A. *et al.* The metabolic syndrome. Endocrine reviews 29, 777–822 (2008). 18971485 10.1210/er.2008-0024 PMC5393149

[b7] Risstad H. *et al.* Five-year outcomes after laparoscopic gastric bypass and laparoscopic duodenal switch in patients with body mass index of 50 to 60: a randomized clinical trial. JAMA 150, 352–361 (2015). 10.1001/jamasurg.2014.3579 25650964

[b8] Dwyer T. *et al.* Cohort Profile: the international childhood cardiovascular cohort (i3C) consortium. International journal of epidemiology 42, 86–96 (2013). 22434861 10.1093/ije/dys004 PMC3600617

[b9] Franks P. W. *et al.* Childhood obesity, other cardiovascular risk factors, and premature death. N Engl J Med 362, 485–493 (2010). 20147714 10.1056/NEJMoa0904130 PMC2958822

[b10] Charakida M. *et al.* Childhood obesity and vascular phenotypes: a population study. Journal of the American College of Cardiology 60, 2643–2650 (2012). 23177297 10.1016/j.jacc.2012.08.1017

[b11] Liang Y. *et al.* Childhood obesity affects adult metabolic syndrome and diabetes. Endocrine 60, 87–92 (2015). 25754912 10.1007/s12020-015-0560-7

[b12] Marini M. A. *et al.* Metabolically healthy but obese women have an intermediate cardiovascular risk profile between healthy nonobese women and obese insulin-resistant women. Diabetes Care 30, 2145–2147 (2007). 17507694 10.2337/dc07-0419

[b13] Karelis A. D. Metabolically healthy but obese individuals. Lancet 372, 1281–1283 (2008). 18929889 10.1016/S0140-6736(08)61531-7

[b14] Karelis A. D. *et al.* The metabolically healthy but obese individual presents a favorable inflammation profile. The Journal of clinical endocrinology and metabolism 90, 4145–4150 (2005). 15855252 10.1210/jc.2005-0482

[b15] Stefan N. *et al.* Identification and characterization of metabolically benign obesity in humans. Arch Intern Med 168, 1609–1616 (2008). 18695074 10.1001/archinte.168.15.1609

[b16] Arnlov J., Ingelsson E., Sundstrom J. & Lind L. Impact of body mass index and the metabolic syndrome on the risk of cardiovascular disease and death in middle-aged men. Circulation 121, 230–236 (2010). 20038741 10.1161/CIRCULATIONAHA.109.887521

[b17] Kuk J. L. & Ardern C. I. Are metabolically normal but obese individuals at lower risk for all-cause mortality? Diabetes care 32, 2297–2299 (2009). 19729521 10.2337/dc09-0574 PMC2782994

[b18] McLaughlin T., Abbasi F., Lamendola C. & Reaven G. Heterogeneity in the prevalence of risk factors for cardiovascular disease and type 2 diabetes mellitus in obese individuals: effect of differences in insulin sensitivity. Arch Intern Med 167, 642–648 (2007). 17420421 10.1001/archinte.167.7.642

[b19] Vukovic R. *et al.* Insulin-sensitive obese children display a favorable metabolic profile. European journal of pediatrics 172, 201–206 (2013). 23090660 10.1007/s00431-012-1867-5

[b20] Arnlov J., Sundstrom J., Ingelsson E. & Lind L. Impact of BMI and the metabolic syndrome on the risk of diabetes in middle-aged men. Diabetes Care 34, 61–65, (2011). 20852030 10.2337/dc10-0955 PMC3005442

[b21] St-Pierre A. C. *et al.* Insulin resistance syndrome, body mass index and the risk of ischemic heart disease. CMAJ 172, 1301–1305, (2005). 15883404 10.1503/cmaj.1040834 PMC557100

[b22] Meigs J. B. *et al.* Body mass index, metabolic syndrome, and risk of type 2 diabetes or cardiovascular disease. The Journal of clinical endocrinology and metabolism 91, 2906–2912 (2006). 16735483 10.1210/jc.2006-0594

[b23] Weiss R. *et al.* Obesity and the metabolic syndrome in children and adolescents. N Engl J Med 350, 2362–2374 (2004). 15175438 10.1056/NEJMoa031049

[b24] Sinaiko A. R. *et al.* Relation of body mass index and insulin resistance to cardiovascular risk factors, inflammatory factors, and oxidative stress during adolescence. Circulation 111, 1985–1991 (2005). 15837953 10.1161/01.CIR.0000161837.23846.57

[b25] Srinivasan S. R., Myers L. & Berenson G. S. Predictability of childhood adiposity and insulin for developing insulin resistance syndrome (syndrome X) in young adulthood: the Bogalusa Heart Study. Diabetes 51, 204–209 (2002). 11756342 10.2337/diabetes.51.1.204

[b26] Singh A. S., Mulder C., Twisk J. W., van Mechelen W. & Chinapaw, M. J. Tracking of childhood overweight into adulthood: a systematic review of the literature. Obesity reviews 9, 474–488 (2008). 18331423 10.1111/j.1467-789X.2008.00475.x

[b27] Johannsson E., Arngrimsson S. A., Thorsdottir I. & Sveinsson T. Tracking of overweight from early childhood to adolescence in cohorts born 1988 and 1994: overweight in a high birth weight population. International journal of obesity 30, 1265–1271 (2006). 16491112 10.1038/sj.ijo.0803253

[b28] Lloyd L. J., Langley-Evans S. C. & McMullen S. Childhood obesity and risk of the adult metabolic syndrome: a systematic review. International journal of obesity 36, 1–11 (2012). 22041985 10.1038/ijo.2011.186 PMC3255098

[b29] Morrison J. A., Friedman L. A., Wang P. & Glueck C. J. Metabolic syndrome in childhood predicts adult metabolic syndrome and type 2 diabetes mellitus 25 to 30 years later. The Journal of pediatrics 152, 201–206 (2008). 18206689 10.1016/j.jpeds.2007.09.010

[b30] Shulman G. I. Ectopic fat in insulin resistance, dyslipidemia, and cardiometabolic disease. The New England journal of medicine 371, 1131–1141 (2014). 25229917 10.1056/NEJMra1011035

[b31] Magnussen C. G. *et al.* A diagnosis of the metabolic syndrome in youth that resolves by adult life is associated with a normalization of high carotid intima-media thickness and type 2 diabetes mellitus risk: the Bogalusa heart and cardiovascular risk in young Finns studies. Journal of the American College of Cardiology 60, 1631–1639 (2012). 23021330 10.1016/j.jacc.2012.05.056

[b32] Weiss R. *et al.* The “obese insulin-sensitive” adolescent: importance of adiponectin and lipid partitioning. The Journal of clinical endocrinology and metabolism 90, 3731–3737 (2005). 15797955 10.1210/jc.2004-2305

[b33] Fabbrini E. *et al.* Metabolically normal obese people are protected from adverse effects following weight gain. The Journal of clinical investigation 125, 787–795 (2015). 25555214 10.1172/JCI78425 PMC4319438

[b34] Roberts R. *et al.* Markers of de novo lipogenesis in adipose tissue: associations with small adipocytes and insulin sensitivity in humans. Diabetologia 52, 882–890 (2009). 19252892 10.1007/s00125-009-1300-4

[b35] Kursawe R. *et al.* Cellularity and adipogenic profile of the abdominal subcutaneous adipose tissue from obese adolescents: association with insulin resistance and hepatic steatosis. Diabetes 59, 2288–2296 (2010). 20805387 10.2337/db10-0113 PMC2927952

[b36] Berenson G. S., *et al.* III. Anthropometry, V Blood Pressure. In Cardiovascular Risk Factors in Children: The Early Natural History of Atherosclerosis and Essential Hypertension (eds Andrews C., Hester H. E.). New York, NY:Oxford University Press, 127–47, 217–86 (1980).

[b37] Elkasabany A. M., Urbina E. M., Daniels S. R. & Berenson G. S. Prediction of adult hypertension by K4 and K5 diastolic blood pressure in children: the Bogalusa Heart Study. The Journal of pediatrics 132, 687–692 (1998). 9580771 10.1016/s0022-3476(98)70361-0

[b38] Grundy S. M. *et al.* Diagnosis and management of the metabolic syndrome: an American Heart Association/National Heart, Lung, and Blood Institute Scientific Statement. Circulation 112, 2735–2752 (2005). 16157765 10.1161/CIRCULATIONAHA.105.169404

