# Supplementary material for: Long-term Impact of Childhood Adiposity on Adult Metabolic Syndrome Is Modified by Insulin Resistance: The Bogalusa Heart Study
Source: Sci Rep. 2015 Dec 7;5:17885. doi: 10.1038/srep17885 (PMC4671136; doi:10.1038/srep17885)
Supplement: Supplementary Information [file srep17885-s1.doc]

**Long-term Impact of Childhood Adiposity on Adult Metabolic Syndrome Is Modified by Insulin Resistance: The Bogalusa Heart Study**

Huijie Zhang^1,2^*, Tao Zhang^2,3^*, Shengxu Li^2^, Ying Li^4^, Azad Hussain^2^, Camilo Fernandez^2^, Emily Harville^2^, Lydia A. Bazzano^2^, Jiang He^2^, Wei Chen^2^

**Supplement Table S1.** Odds ratio of childhood HOMA for adult overweight/obesity, hyperglycemia, high blood pressure and dyslipidemia, adjusted for baseline age, race, gender and follow-up years.

| Dependent variables | Independent variable: HOMA (log-transformed) | | |
| --- | --- | --- | --- |
|  | OR | 95%CI | P |
| Overweight/obesity ^a^ | 1.70 | 1.50-1.92 | <0.001 |
| Hyperglycemia ^b^ | 1.35 | 1.13-1.61 | 0.001 |
| High blood pressure ^c^ | 1.28 | 1.12-1.46 | <0.001 |
| Dyslipidemia ^d^ | 1.23 | 1.11-1.38 | <0.001 |

HOMA=homeostasis model assessment of insulin resistance; WC=waist circumference; HDL-C=high-density lipoprotein cholesterol; OR=odds ration; CI=confidence interval

a, Overweight/obesity was defined as WC ≥102 cm for men and ≥88 cm for women.

b, Hyperglycemia was defined as fasting glucose≥100 mg/dL or taking antidiabetic medication

c, High blood pressure was defined as blood pressure≥130/85 mmHg or taking antihypertensive medication

d, Dyslipidemia was defined as triglycerides≥150 mg/dL, or HDL-C< 40 mg/dL for men and <50 mg/dL for women, or taking cholesterol lowering medication

**Supplement Table S2.** Association between childhood insulin-resistant and insulin-sensitive adiposity and adult MetS, adjusted for covariates

|  | Using the BMI↑/Insulin↓ group as a reference^a^ | | | | | | | | |
| --- | --- | --- | --- | --- | --- | --- | --- | --- | --- |
|  | Whites (n=422) | | | Blacks (n=231) | | | Total (n=653) | | |
|  | OR | 95%CI | P | OR | 95%CI | P | OR | 95%CI | P |
| Black race | --- | --- | --- | --- | --- | --- | 0.67 | 0.44~1.00 | 0.054 |
| Female sex | 0.59 | 0.38~0.92 | 0.021 | 0.56 | 0.27~1.16 | 0.12 | 0.58 | 0.40~0.85 | 0.005 |
| Childhood age | 1.07 | 1.01~1.14 | 0.033 | 1.15 | 1.05~1.26 | 0.002 | 1.10 | 1.05~1.16 | <0.001 |
| Follow-up years | 1.13 | 1.09~1.17 | <0.001 | 1.16 | 1.11~1.22 | <0.001 | 1.14 | 1.11~1.17 | <0.001 |
| BMI↑/Insulin↑^a^ | 1.68 | 1.05~2.72 | 0.032 | 1.20 | 0.57~2.56 | 0.636 | 1.53 | 1.03~2.29 | 0.036 |
|  | | | | | | | | | |
|  | Using the BMI↑/Glucose↓ group as a reference^a^ | | | | | | | | |
|  | Whites (n=416) | | | Blacks (n=226) | | | Total (n=642) | | |
|  | OR | 95%CI | P | OR | 95%CI | P | OR | 95%CI | P |
| Black race | --- | --- | --- | --- | --- | --- | 0.71 | 0.45~1.06 | 0.094 |
| Female sex | 0.57 | 0.37~0.89 | 0.014 | 0.70 | 0.34~1.45 | 0.335 | 0.61 | 0.42~0.89 | 0.01 |
| Childhood age | 1.09 | 1.03~1.17 | 0.006 | 1.16 | 1.06~1.27 | 0.001 | 1.12 | 1.06~1.17 | <0.001 |
| Follow-up years | 1.12 | 1.09~1.16 | <0.001 | 1.16 | 1.11~1.23 | <0.001 | 1.14 | 1.11~1.17 | <0.001 |
| BMI↑/Glucose↑^a^ | 0.76 | 0.49~1.19 | 0.238 | 0.91 | 0.44~1.87 | 0.788 | 0.79 | 0.54~1.15 | 0.220 |

^a^, childhood adiposity and insulin resistance were defined as BMI and insulin or glucose above 55th percentile, respectively.
